# Iatrogenic Rupture of Saline Breast Implant Following Robotic-Assisted Lung Surgery

**DOI:** 10.7759/cureus.35236

**Published:** 2023-02-20

**Authors:** Joyce Y Cui, Tawee Tanvetyanon

**Affiliations:** 1 Department of Medical Education, University of South Florida Morsani College of Medicine, Tampa, USA; 2 Department of Thoracic Oncology, Moffitt Cancer Center, Tampa, USA

**Keywords:** robotic-assisted surgery, iatrogenic, rupture, minimally invasive surgery, breast augmentation, breast implant

## Abstract

Breast implants are prevalent in the United States. Patients with breast implants may be susceptible to iatrogenic implant rupture during cardiothoracic surgery. To our knowledge, however, this complication has never been described following robot-assisted thoracic surgery (RATS). We described a patient who developed a rupture of a saline breast implant after undergoing a robot-assisted left lower lobectomy. We hypothesized that a tear to the breast implant may have occurred either during the extraction of one of the surgical ports or during the forceful extraction of the resected specimen. This case highlights another potential complication of minimally invasive thoracic surgery. For patients with breast implants undergoing thoracic RATS, extra care should be made to the site of port placement and the avoidance of excessive force during surgery.

## Introduction

Breast implants are common in the United States. In 2021, over 360,000 breast augmentation procedures were performed using breast implants [1]. Modern breast implants which consist of either saline or cohesive gel can last for decades; however, an unexpected rupture may occur. For instance, rupture rates of 3% to 5% at three years and 7% to 10% at 10 years have been reported from a 10-year prospective study of saline breast implants [2]. Rupture may occur spontaneously due to degradation of the implant shell material, or it may occur as a result of trauma or excessive pressure on the implant such as during mammography [2]. For saline implants, the rupture will result in deflation followed by saline filling being absorbed into blood circulation.

Cardiothoracic surgery is another known cause of breast implant rupture. In a retrospective study of 78 patients with breast implants who underwent cardiac surgery or pacemaker placement, four patients (5%) developed rupture within 90 days following the surgery [3]. The authors found that patients who underwent median sternotomy were at a higher risk than others. Some cardiac surgeons even recommend the removal of breast implants for optimal chest wall exposure and replace the same implant after surgery [4]. For thoracic surgery, however, no study has been available to help estimate the incidence of iatrogenic breast implant rupture. To date, only a small number of case reports have been published to describe this possible complication after video-assisted thoracoscopic surgery (VATS) [5,6] or after chest tube insertion [7]. To our knowledge, there has been no report of breast implant rupture following a robotic-assisted thoracic surgery (RATS), an increasingly adopted, minimally invasive surgical approach.

We recently cared for a patient who developed a unilateral breast implant rupture following RATS for left lower lobectomy. The rupture of breast implants results in serious aesthetical consequences for the affected patient. The patient may be psychologically traumatized. It can be difficult to restore the appearance to the pre-rupture state. In addition, legal consequences may follow. Given that there will be many more patients with breast implants undergoing RATS in the future, a detailed analysis of this case may help to prevent future occurrences.

## Case presentation

**Figure 1:**
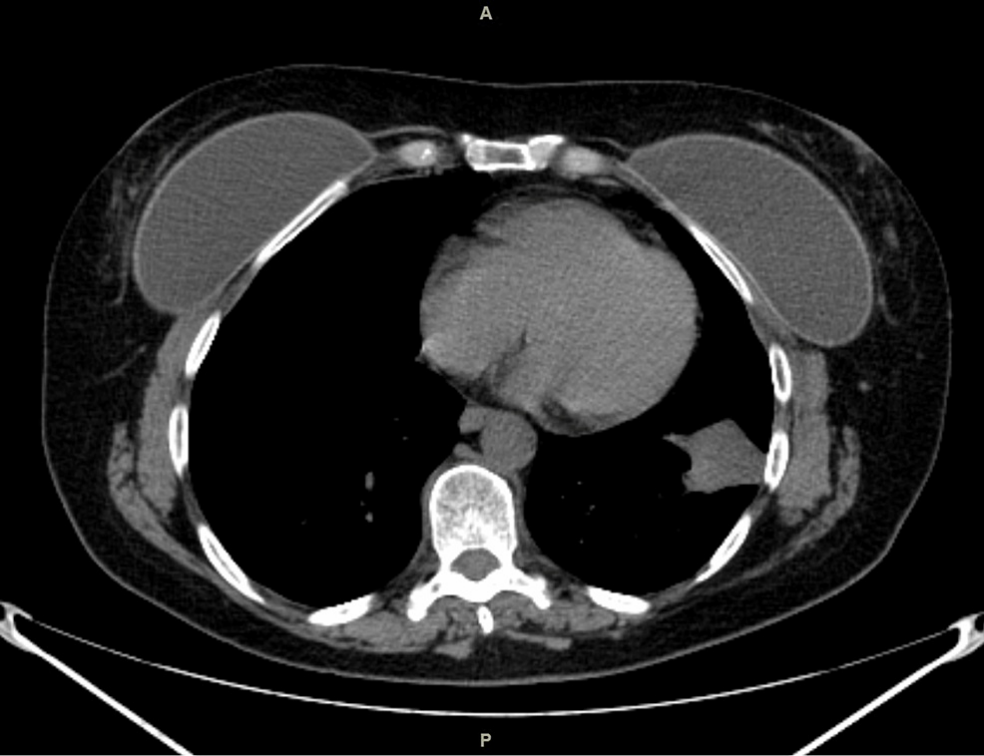
Computerized tomography of the chest demonstrating a left lower lobe lung mass before surgery

A 59-year-old female patient presented with persistent coughs. Chest computerized tomography (CT) scan revealed a mass in the left lower lobe (Figure 1). A biopsy confirmed adenocarcinoma. Her pulmonary function was adequate, and her body mass index was 27 kg/m^2^. The patient had no significant medical comorbidity except for the fact that she underwent bilateral aesthetic breast augmentation with saline implants 12 years ago.

She underwent a robot-assisted left lower lobectomy. During the procedure, the patient was placed in a lateral decubitus position with the right chest up. Four ports were placed: an 8-mm incision was made at the 9th intercostal space in line with the tip of the scapula to accommodate an 8-mm 30-degree thoracoscope for the Intuitive da Vinci Xi® robotic surgical system (Sunnyvale, CA, USA), another 8-mm port was placed anteriorly in the 4th intercostal space, another 12-mm port was placed in the same intercostal space posterior to the camera, and finally, the other 12-mm port was placed in line with a 35-mm incision along the major fissure in the anterior axillary line. Multi-level intercostal nerve block via thoracoscopic guidance was performed using bupivacaine and epinephrine. An extra-small wound protector was placed in the 35-mm incision. During this procedure, her left lower lobe bronchus was identified and dissected along with the inferior pulmonary ligament lymph node and the lymphatic tissue at the aorticopulmonary window. Subsequently, all resected specimens were retrieved through the 35-mm incision. There were no intraoperative or immediate post-operative complications. The patient was discharged from the hospital two days afterward. The final pathology examination demonstrated a 7-cm adenocarcinoma of the left lower lobe with negative lymph nodes, consistent with stage T3N0.

**Figure 2:**
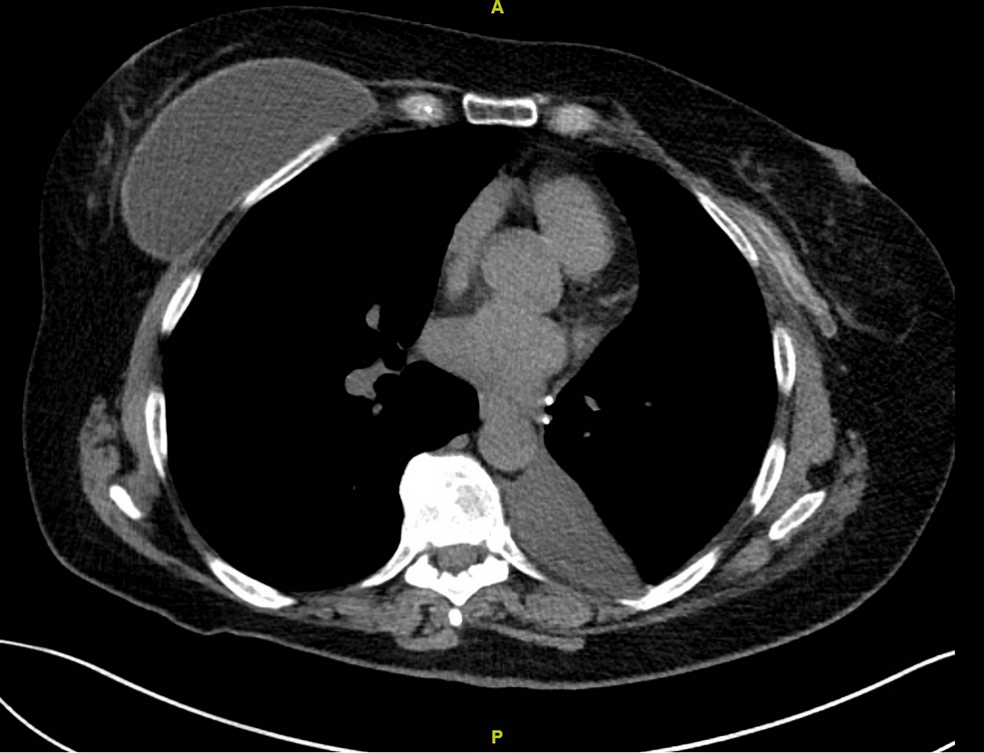
Computerized tomography of the chest demonstrating deflation of left breast implant

**Figure 3:**
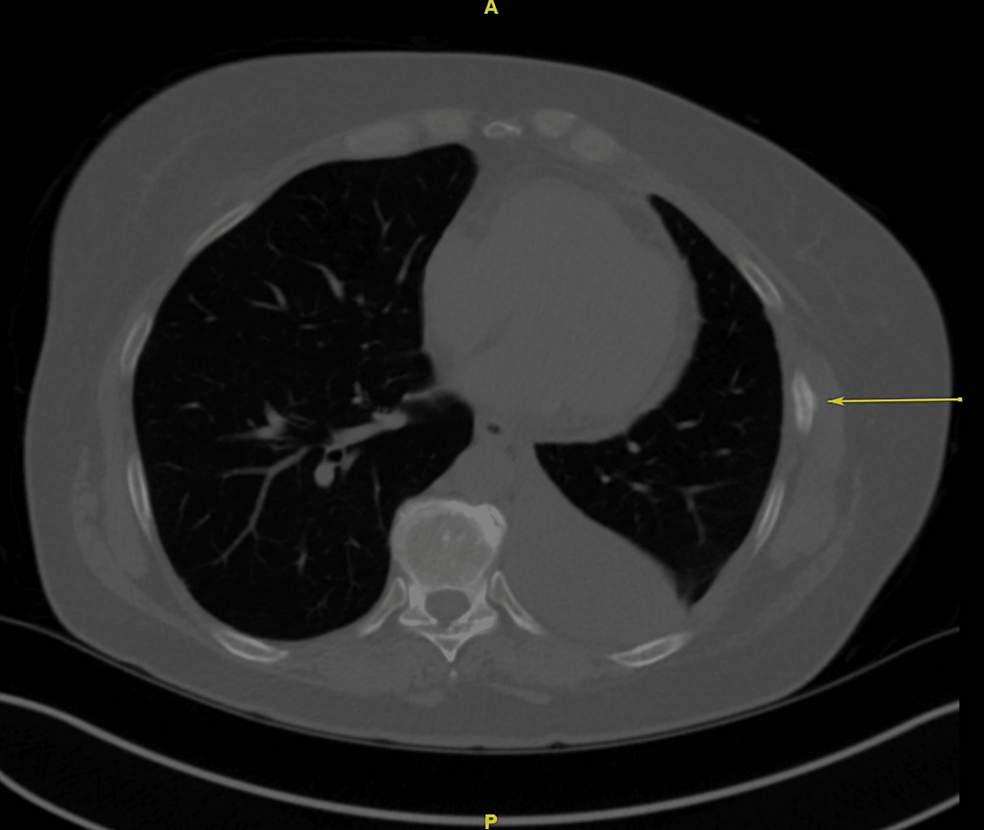
Computerized tomography of the chest demonstrating non-displaced rib fracture (arrow)

One month after surgery, the patient reported that she felt a deflation of her left breast. During the examination, a deflation of the breast along with redness in the dependent portion of her lower anterior chest was evident. Chest CT scan demonstrated the deflation (Figure 2) as well as healed, non-displaced fractures of the anterior left 7th rib (Figure 3) and posterior 9th rib.

During her visit to a medical oncologist for adjuvant chemotherapy, she appeared distraught. The patient wanted to have her breast implant replaced before adjuvant chemotherapy. She was occupied with getting her new implants and missed the time window for adjuvant chemotherapy. Five months after RATS, both breast implants were removed through a bilateral capsulectomy. The right breast implant was found intact as a 450-ml saline implant. The left breast implant was found ruptured and folded on itself. A new pair of saline implants were inserted.

**Figure 4:**
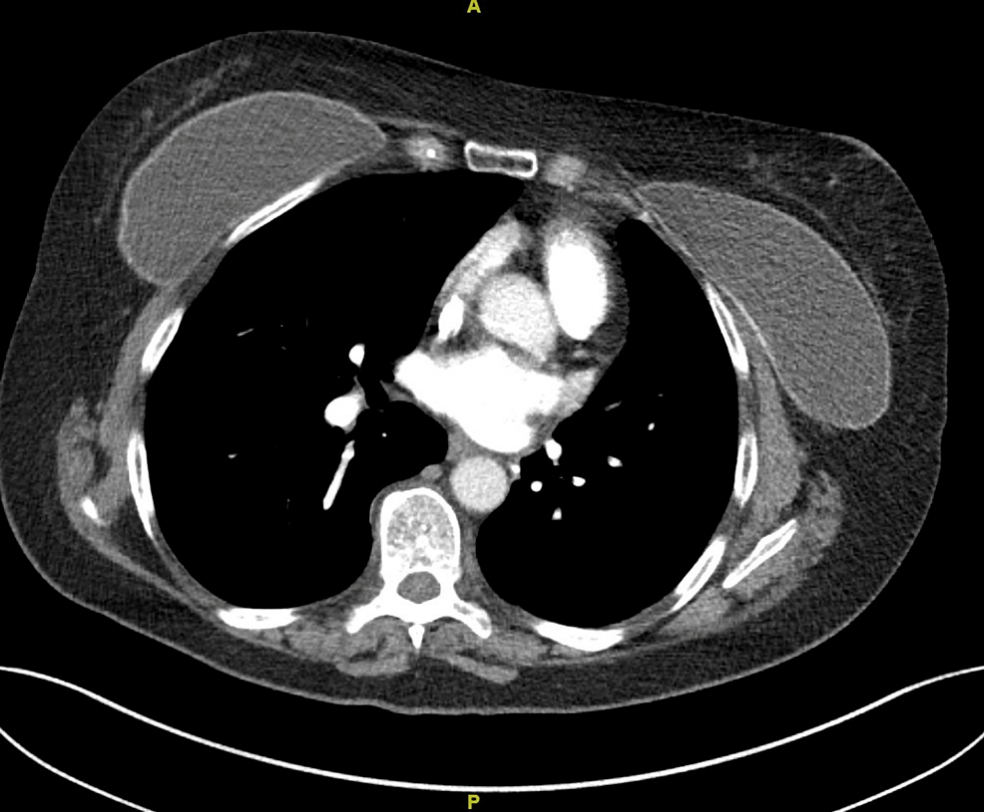
Computerized tomography of the chest after a replacement of breast implants

Six months after this procedure, the patient reported no complications, and a chest CT showed no evidence of lung cancer recurrence (Figure 4).

## Discussion

We described a patient who developed a rupture of a saline breast implant following RATS for lobectomy. The deflation occurred gradually over the course of one month following RATS. The patient underwent a procedure for the replacement of both breast implants about five months later. She missed an opportunity for adjuvant chemotherapy as a result of overwhelming grief from the complication.

What could have caused the breast implant to rupture? To our knowledge, this is the only case of breast implant rupture associated with lung cancer surgery at our institution. The thoracic surgeon who performed RATS, in this case, had over 15 years of experience with RATS and there was no trainee involved during the procedure. This complication occurred despite all routine safety standards during RATS being followed. It seems highly unlikely that the implant may have ruptured by itself because it was only 12 years old and the matching right implant was found to be intact. The close temporal relationship between RATS and rupture as well as the left laterality suggest that the rupture was caused by RATS. Our literature search did not find any reports of breast implant rupture associated with RATS. It is possible that this complication is very rare or under-reported. Based on the operative report, we suspect that accidental damage to the breast implant may have occurred during the trocar insertion or extraction of the fourth intercostal spaceport. It is also possible that the rupture may have occurred during the extraction of the surgical specimen which may have been considerably forceful as reflected by multiple rib fractures.

RATS has been increasingly adopted in place of open thoracotomy. Some authors have found it to be associated with lower morbidity, shorter hospital stay, and better quality of life [8]. Complications related to RATS are infrequent. In a study with 1264 patients who underwent RATS for pulmonary resections, 4.3% of patients experienced at least one major complication including pneumonia, prolonged air leak, respiratory distress, and cardiac arrhythmias [9]. Since RATS will likely be performed for many more patients with breast implants in the future, we believe it is important to consider breast implant rupture as another possible complication of RATS. To prevent this, the instrument port should not be placed close to the implant and care should be taken to avoid applying excessive force during the procedure. If force seems necessary to extract a large tumor, alternative strategies or even a consideration for upfront thoracotomy should be considered or discussed with the patient before the surgical decision. Furthermore, for necessary procedures near breast implants, a consultation with a plastic surgeon may be helpful. This strategy has been shown to reduce the incidence of unexpected complications related to breast implants after cardiac surgery [3].

Although this report is the first to suggest a relationship between RATS and the rupture of breast implants, there are important limitations. First, we cannot completely rule out other causes of rupture such as trauma which may have occurred at any time point between the baseline chest CT and the deflation. However, the patient did not report any trauma or mammography. Second, we did not have available data from a formal shell inspection of the retrieved implant. A formal shell inspection may reveal characteristics of the rupture site, providing clues regarding the nature of the damage. However, given the expected small damage resulting in a slow leakage, even a formal shell inspection may not be informative.

## Conclusions

In summary, our experience with this case suggests that breast implant rupture is another potential complication associated with RATS pulmonary resection. We hope that this case report will help other clinicians further understand potential causes for breast implant rupture during a RATS pulmonary resection and how to best avoid this complication. Ultimately, being able to do so will lead to a better continuation of treatment and minimize emotional distress for the patient.
